# Editorial: Recent Advances in Doppler Signal Processing and Modeling Techniques for Fetal Monitoring

**DOI:** 10.3389/fphys.2018.00691

**Published:** 2018-06-05

**Authors:** Ahsan H. Khandoker, Faezeh Marzbanrad, Yoshitaka Kimura

**Affiliations:** ^1^Department of Biomedical Engineering, Khalifa University, Abu Dhabi, United Arab Emirates; ^2^Department of Electrical and Computer Engineering, Monash University, Clayton, VIC, Australia; ^3^Graduate School of Medicine, Tohoku University, Sendai, Japan

**Keywords:** fetal Doppler ultrasound, fetal electrocardiogram (FECG), fetal phonocardiogram, fetal monitoring, fetal screening

The intersection of perinatal medicine and biomedical engineering is an emerging scientific research area. Understanding pathophysiological process of fetal development and improving the technologies used in perinatal diagnosis and intervention in clinical settings are vital for monitoring fetal well-being reliably. The major challenges in fetal Doppler, acoustic and electrical signal processing techniques which are often used in obstetrical instrumentations are poor specificity with high false positive rates and strong non-stationarities in abdominal derived signals. Therefore, more research is needed to explore the untapped potentials of abdominal sensor or lead based fetal signal (Doppler, ECG, Phonogram etc.) analyses and modeling.

In this research topic, selected emerging technologies in abdominal derived fetal signal processing for screening of fetal development and well-being are published. These papers include reviews, commentaries and technical contributions on: (1) challenges in fetal cardiac signal processing techniques; (2) nonlinear signal processing techniques in fetal Doppler signals; (3) novel techniques for detection of fetal heart sounds; (4) quality assessment of Doppler signals; (5) abdominal fetal electrocardiography technique and its application on growth restricted fetuses; (6) comparative study on fetal heart rates; (7) application of fetal Doppler ultrasound and fetal electrocardiography in pregnant animal model (mouse).

The guest editors of this research topic have accepted 10 very high-quality submissions for inclusion in this special issue. The key difference between this issue and contemporary fetal physiology related literature is that this research topic summarizes additional insights into the physiological link between physiologically understandable mathematical indices of fetal signals and the developing cardiovascular functions in fetal health and compromises. The summary of the research papers published in this topic is given below within three main categories.

## Fetal cardiogenic signals

Cardiogenic such as mechanical, auscultation and electrical activities of fetal heart are acquired by abdominal Doppler, Phonogram and ECG technologies.

Frasch et al. (2017) in a general commentary shared the experience from a randomized controlled trial with computerized interpretation of fetal heart rate during labor (INFANT) collaborative group. They proposed the importance of new modalities of direct acquisition of predictive information about the fetal brain and heart so that a variety of fetal compromises could be prevented.

Alnuaimi et al. reviewed the most recent progress in fetal cardiac Doppler signal processing research. The review focuses on the shortcomings and advantages, which helps in understanding fetal Doppler cardiogram signal processing methods, and the related Doppler signal analysis procedures, by providing valuable clinical information, with a set of recommendations for future research directions.

One of the challenges of fetal monitoring techniques, such as Doppler ultrasound, is the susceptibility to noise affecting the signal quality, and subsequently the accuracy and reliability of the derived metrics (Stroux and Clifford, 2014; Marzbanrad et al., 2015; Valderrama et al., 2018). Valderrama et al. proposed an interesting study on how to assess the quality of Doppler signals, which are inherently non-stationary, and highly movement sensitive signals. The proposed template based method introduced a new signal quality index, which is validated on recordings from a low cost Doppler probe. This novel method might be useful in resource-poor settings or when operated by non-skilled operators.

Koutsiana et al. demonstrated a Wavelet Transform-based method combined with the Fractal Dimension to detect fetal heart sounds from abdominal phonogram signals. Fetal phonography is popular with low-cost option in many developing countries in the world.

Li et al. (2017) demonstrated an efficient method of extracting fetal and maternal ECG signals from two channels, which are attached on abdominal leads. The proposed method was then validated on a non-invasive fECG database in PhysioNet. The proposed technique might be useful in ambulatory monitoring of fetal vital signals at home.

Velayo et al. then showed a real application of abdominal lead based fetal ECG in growth-restricted fetuses. The results of the study confirmed that both QT and QTc intervals were significantly prolonged in growth-restricted fetuses as compared to a control group. It clearly highlights the potential of fetal ECG as a potential clinical screening tool to aid diagnosis and management of compromised fetuses.

## Fetal heart rates and valve timing intervals

Fetal heart rate (FHR) monitoring by Doppler based Cardiotocography (CTG) in the third trimester is a commonly established method to identify fetal compromises (Sandmire and DeMott, 1998). However, sometimes abnormal variability in FHR may not necessarily represent the fetus in distress (Murphy et al., 1991; Vincent et al., 1991). Doppler Ultrasound can provide more information on fetal wellbeing and development beyond the FHR. Using FECG as a reference, automated techniques were proposed in order to identify fetal cardiac valve motion from Doppler signals, providing new measures of mechanical and electromechanical activity of the fetal heart (Marzbanrad et al., 2014, 2016). It provides systolic time intervals (STI) of the fetal cardiac cycle, which have been analyzed by several authors in the past and showed differentiation of fetuses with a variety of perinatal problems (Organ et al., 1980; Koga et al., 2001; Khandoker et al., 2009).

Al-Angari et al. presented a hybrid Empirical Mode Decomposition-Kurtosis method to estimate beat-to-beat fetal heart rate from continuous Doppler signals and then compare with the same from abdominal lead fetal ECG signals.

Jezewski et al. then showed the evidence of equivalence of those two methods (Doppler signals and fetal ECG signals) in terms of recognition of classical FHR patterns such as baseline, accelerations/decelerations, short- and long-term variabilities. These findings might be very useful in clinical settings, as Doppler based FHR monitors are commonly used in routine obstetrics check-ups.

Marzbanrad et al. explained the method on how to estimate fetal cardiac valves' timing intervals from Doppler signals and how those parameters are correlated with fetal gestational development. The proposed method could provide new measures for fetal physiological development.

## Animal model in perinatal monitoring research

Funamoto et al. demonstrated how a fetal mouse model could be utilized to investigate the fetal brain hemorrhage in an ischemia/reperfusion model by using ultrasound B-mode imaging. The use of fetal mice is a novel model for future perinatal research in a variety of fetal stress and compromise.

## Concluding remarks

Effective prediction and prevention of fetal stress, compromise or anomalies have now become an emerging research priority. Traditional methods of screening fetal well-being are still popular within the clinical community, however, they demonstrate clear limitations. This research topic briefly presents an overview of original and relevant contributions covering the areas of emerging new signal processing techniques and algorithms enabling early diagnosis of fetal compromises. It is hoped that the proposed technologies and systems could result in improved fetal health management and treatment at the point of need, reduced unnecessary C-section, and the associated economic burden, offering a better quality of early start of life in this world.

## Author contributions

All authors listed have made a substantial, direct and intellectual contribution to the work, and approved it for publication.

### Conflict of interest statement

The authors declare that the research was conducted in the absence of any commercial or financial relationships that could be construed as a potential conflict of interest.
